# Supplementary material for: Xanthomonas citri subsp. citri requires a genus-specific outer membrane protein and TolB to coordinate cell membrane integrity and virulence
Source: Microbiol Spectr. 2025 Jan 16;13(2):e02521-24. doi: 10.1128/spectrum.02521-24 (PMC11792487; doi:10.1128/spectrum.02521-24)
Supplement: Supplemental figure captions — Figure captions for Fig. S1 to S5. [file spectrum.02521-24-s0001.docx]

SUPPLEMENTAL MATERIALS

**FIG S1** Schematic diagram showing the cloned *gum* promoter. A 394-bp *gum* promoter region in front of *gunB* gene was cloned from wild-type 29-1. The locations of primers PgumB.F and PgumB.R are under lined. Predicted core structure of *gum* promoter is shadowed.

**FIG S2** SDS-PAGE analysis of the final eluted proteins in the GST pull-down. The purified GST-OMP*_Xan_* and total proteins of *Xanthomonas citri* subsp. *citri* were co-incubated overnight. The mixture was then subjected to a pull-down assay using the glutathione resin. The proteins that were pulled down were scored by 10% SDS-PAGE electrophoresis by comparison with the negative GST control. GST, glutathione-S-transferase.

**FIG S3** Deletion mutagenesis of *tolB*. (A) Schematic diagram showing the deletion mutagenesis of *tolB*. The locations of primers are underlined. (B) Identification of the deletion mutant of *tolB*. The difference in sizes of the PCR products from the WT *Xcc* 29-1 (1899 bp) and mutant (756 bp) was revealed using primers tolB1.F and tolB2.R. M, DNA marker DL2000; Lane 1, WT; Lane 2, Mutant. WT, wild type.

**FIG S4** *hrpG* and *hrpX* promoter activities in Δ*OMP_xan_.* The activities of GUS were quantified in Δ*OMP_xan_* that harbored P*hrpG*-GUS or P*hrpX*-GUS fusions cultured in the *hrp*-inducing medium XVM2. The GUS activity was measured with *p*-nitrophenol-β-D-glucuronide as substrate and counted as nmol product min^-1^ OD^-1^. GUS, β-glucuronidase.

**FIG S5** Phenotypic analysis of the *OMP_xan_* mutant of *Xanthomonas campestris* pv. *campestris.* (a) Reduced extracellular polysaccharide production of the *OMP_xan_* mutant on NB plates. (b) Black rot caused by Δ*OMP_xan_* of *Xanthomonas campestris* pv. *campestris.* The cell suspension (OD_600_=0.3) was inoculated on wild cabbage (*Brassica oleracea*) by the leaf-cutting method. The phenotype was recorded at 7 d post-inoculation.
