# Supplementary figures and images for: Xanthomonas citri subsp. citri requires a genus-specific outer membrane protein and TolB to coordinate cell membrane integrity and virulence
Source: Microbiol Spectr. 2025 Jan 16;13(2):e02521-24. doi: 10.1128/spectrum.02521-24 (PMC11792487; doi:10.1128/spectrum.02521-24)

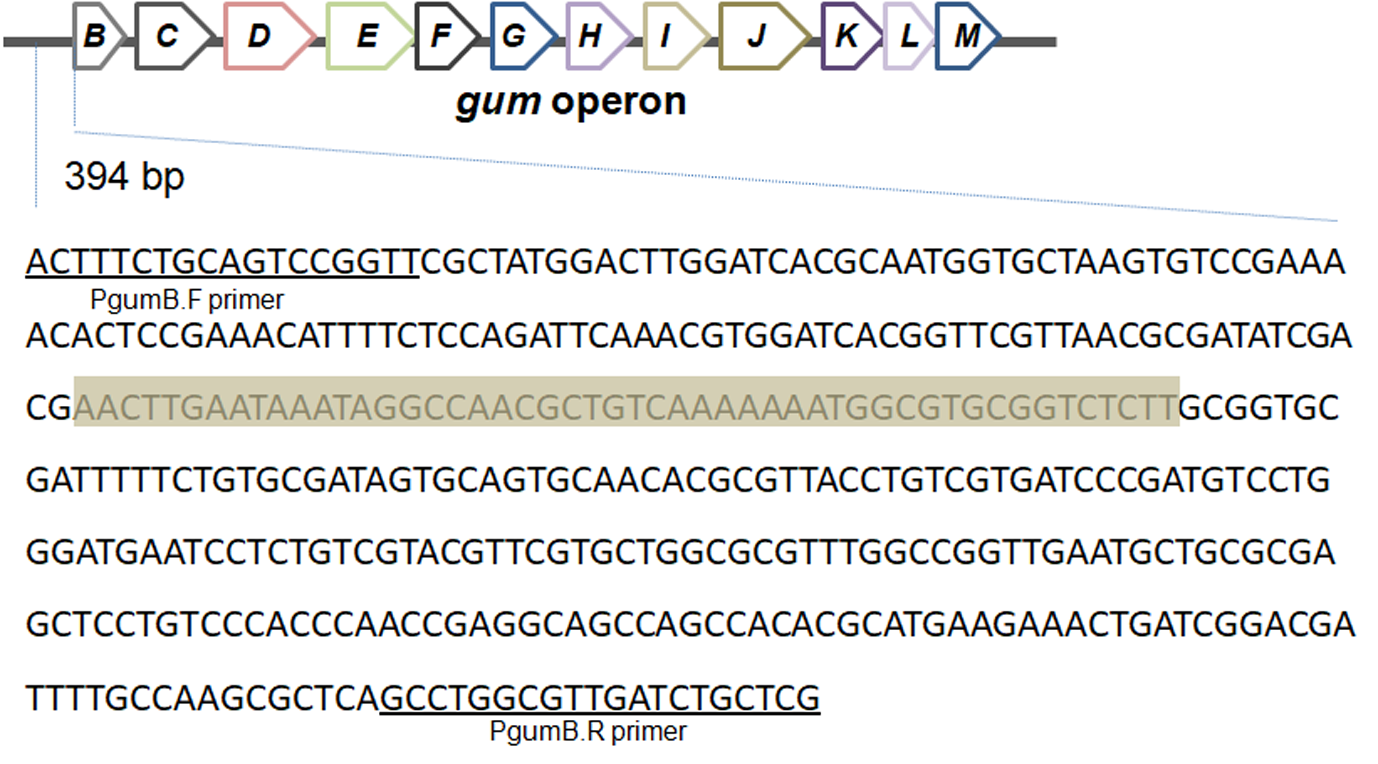

Supplement: Figure S1 — Schematic diagram showing the cloned gum promoter. [file spectrum.02521-24-s0002.tif]

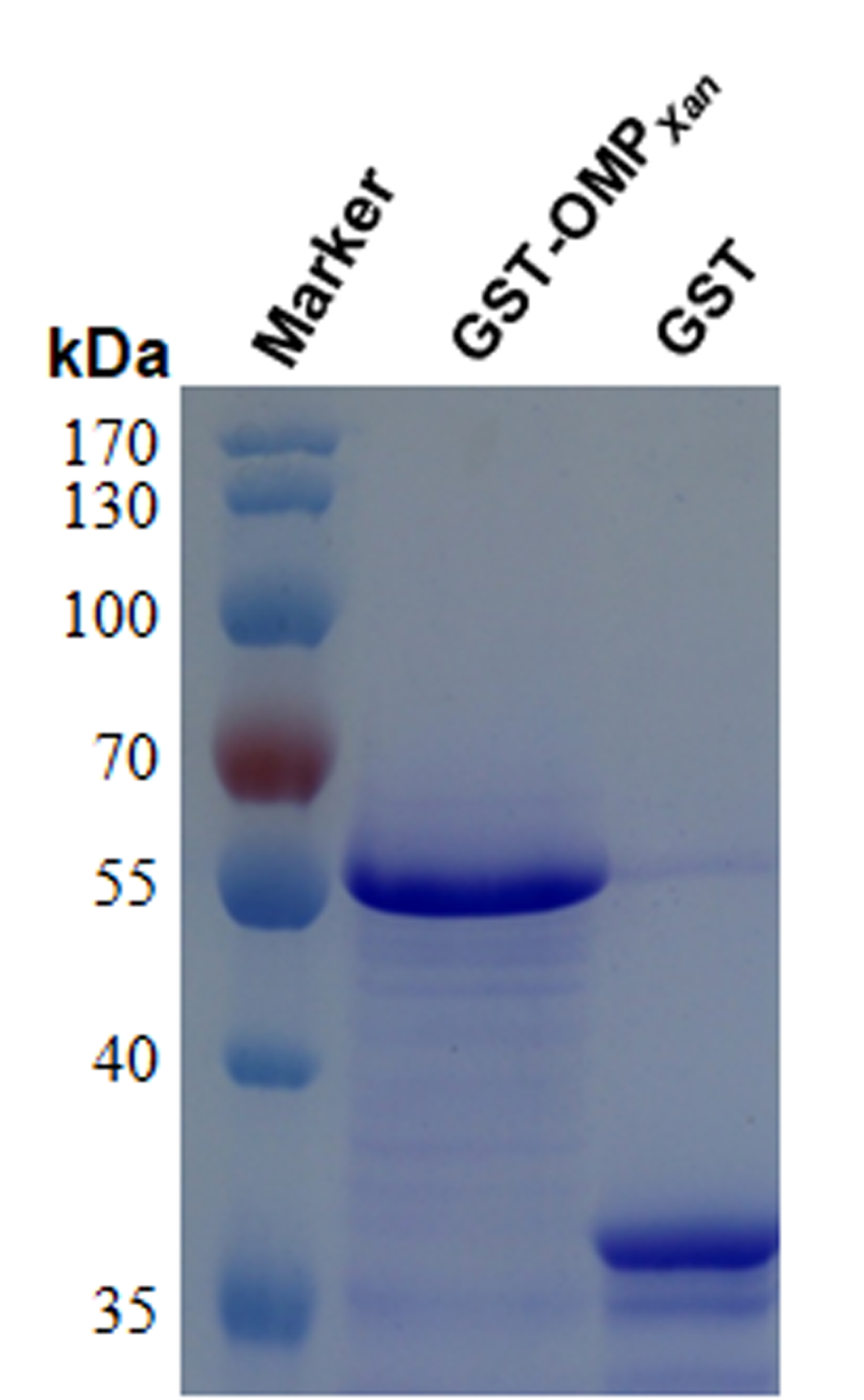

Supplement: Figure S2 — SDS-PAGE analysis of the final eluted proteins in the GST pull-down. [file spectrum.02521-24-s0003.tif]

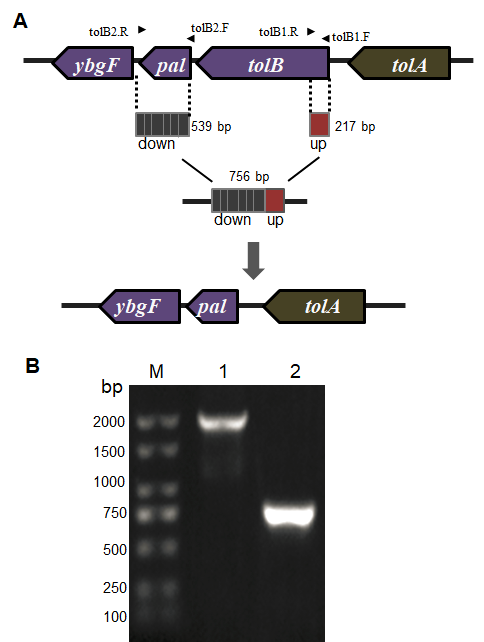

Supplement: Figure S3 — Deletion mutagenesis of tolB. [file spectrum.02521-24-s0004.tif]

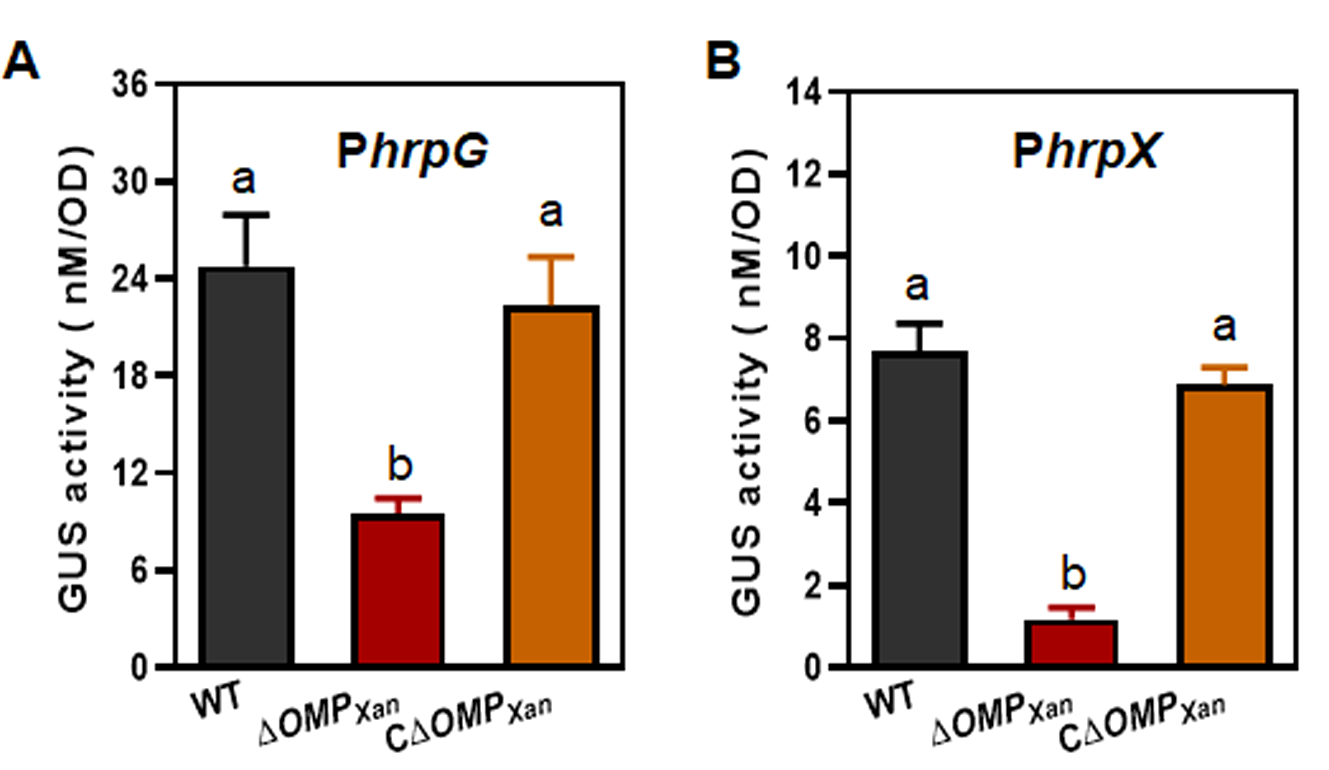

Supplement: Figure S4 — hrpG and hrpX promoter activities in ΔOMPxan. [file spectrum.02521-24-s0005.tif]

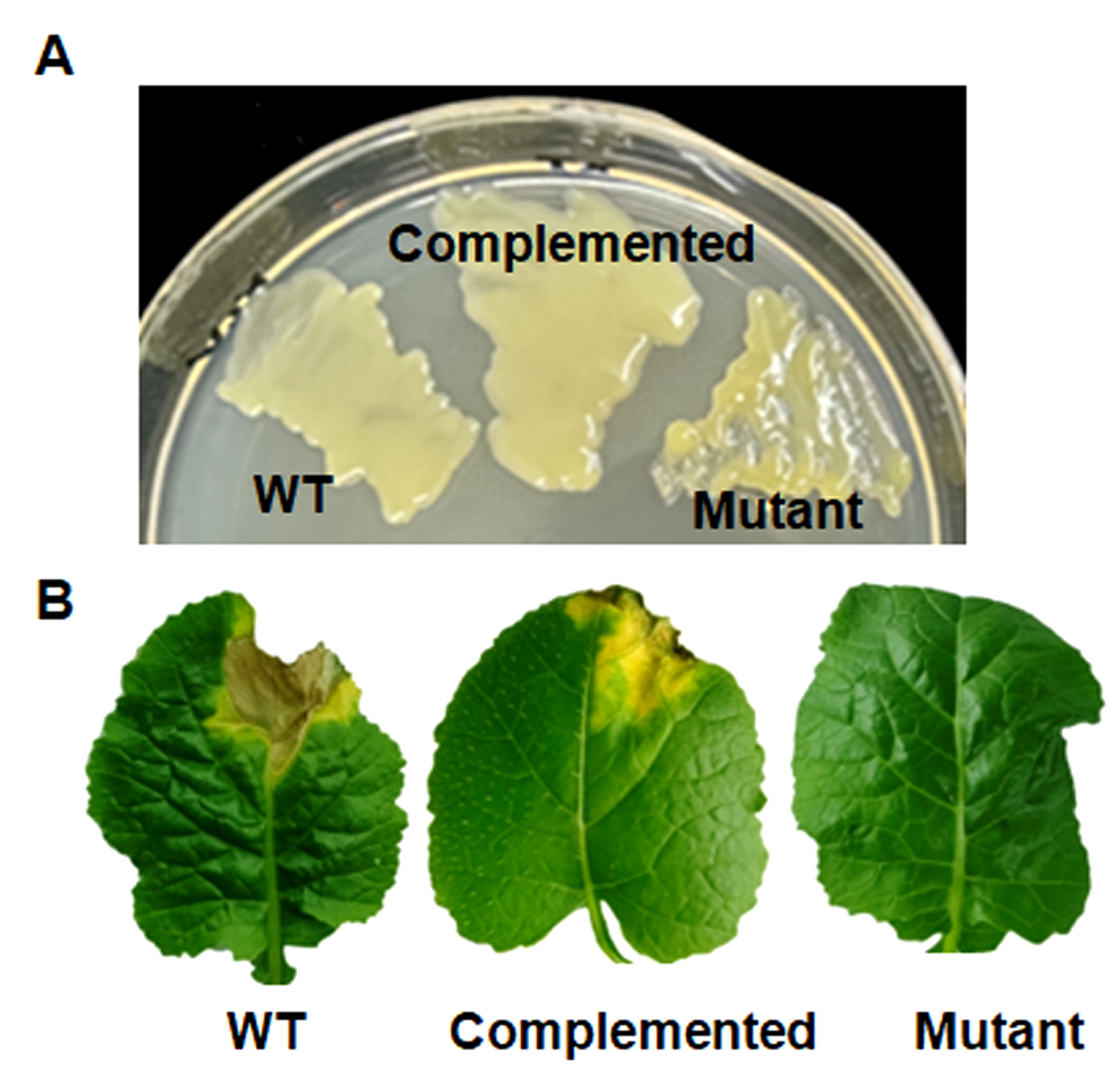

Supplement: Figure S5 — Phenotypic analysis of the OMPxan mutant of Xanthomonas campestris pv. campestris. [file spectrum.02521-24-s0006.tif]
