# Supplementary material for: Xanthomonas citri subsp. citri requires a genus-specific outer membrane protein and TolB to coordinate cell membrane integrity and virulence
Source: Microbiol Spectr. 2025 Jan 16;13(2):e02521-24. doi: 10.1128/spectrum.02521-24 (PMC11792487; doi:10.1128/spectrum.02521-24)
Supplement: Table S1 — Characterized OMPXan-interacting proteins from Xanthomonas citri subsp. citri [file spectrum.02521-24-s0007.docx]

| **Accession** | **Description** | **Gene ID** | | **Mass** | **Score** | **Matches** | **Sequences** | **emPAI** | **Coverage** |
| --- | --- | --- | --- | --- | --- | --- | --- | --- | --- |
|  |  | *Xcc* 306 strain | *Xcc* 29-1 strain |  |  |  |  |  |  |
| B2STJ5 | Protein TolB | XAC3142 | XAC29_15985 | 47113 | 2207 | 111(79) | 27(22) | 6.13 | 68% |
| Q3BWY6 | Elongation factor Tu | XAC0957  XAC0970 | XAC29_04905 XAC29_04845 | 43344 | 1440 | 58(40) | 19(15) | 5.29 | 62% |
| Q8PJ31 | Phosphoglucosamine mutase | XAC2714 | XAC29_13835 | 47408 | 921 | 27(21) | 13(10) | 1.74 | 49% |

Table S1 Characterized OMP*_Xan_*-interacting proteins from *Xanthomonas citri* subsp. *citri*
