# Supplementary material for: Xanthomonas citri subsp. citri requires a genus-specific outer membrane protein and TolB to coordinate cell membrane integrity and virulence
Source: Microbiol Spectr. 2025 Jan 16;13(2):e02521-24. doi: 10.1128/spectrum.02521-24 (PMC11792487; doi:10.1128/spectrum.02521-24)
Supplement: Table S2 — Bacterial strains and plasmids used in this study. [file spectrum.02521-24-s0008.docx]

Table S2. Bacterial strains and plasmids used in this study

| **Strain or plasmid** | **Relevant characteristics** | **Reference** |
| --- | --- | --- |
| **Strains** | | |
| *Xanthomonas citri* subsp. *citri* | | |
| *Xcc* 29-1 | Wild-type strain isolated from *Citrus sinensis* in Jiangxi Province, China | (1) |
| Δ*OMP_Xan_* | A non-polar mutant of *OMP_Xan_* derived from *Xcc* 29-1 | (1) |
| *C*Δ*OMP_Xan_* | Gm^r^, Δ*OMP_Xan_* expressing *OMP_Xan_* harbored in pBBR1MCS-5 | (1) |
| Δ*tolB* | A non-polar mutant of *tolB* derived from *Xcc* 29-1 | This study |
| *C*Δ*tolB* | Gm^r^, Δ*tolB* complemented by pBB-tolB | This study |
| *Xcc* 29-1/P*hrpG*-GUS | Sp^r^, *Xcc* 29-1 carrying pRG960-P*hrpG* | This study |
| *Xcc* 29-1/P*hrpX*-GUS | Sp^r^, *Xcc* 29-1 carrying pRG960-P*hrpX* | This study |
| *Xcc* 29-1/P*gumB*-GUS | Sp^r^, *Xcc* 29-1 carrying pRG960-P*gumB* | This study |
| Δ*OMP_Xan_*/P*hrpG*-GUS | Sp^r^, Δ*OMP_Xan_* carrying pRG960-P*hrpG* | This study |
| Δ*OMP_Xan_*/P*hrpX*-GUS | Sp^r^, Δ*OMP_Xan_* carrying pRG960-P*hrpX* | This study |
| Δ*OMP_Xan_*/P*gumB*-GUS | Sp^r^, Δ*OMP_Xan_* carrying pRG960-P*gumB* | This study |
| CΔ*OMP_Xan_*/P*hrpG*-GUS | Gm^r^Sp^r^, CΔ*OMP_Xan_* carrying pRG960-P*hrpG* | This study |
| CΔ*OMP_Xan_*/P*hrpX*-GUS | Gm^r^Sp^r^, CΔ*OMP_Xan_* carrying pRG960-P*hrpX* | This study |
| CΔ*OMP_Xan_*/P*gumB*-GUS | Gm^r^Sp^r^, CΔ*OMP_Xan_* carrying pRG960-P*gumB* | This study |
| Δ*tolB*/P*hrpG*-GUS | Sp^r^, Δ*tolB* carrying pRG960-P*hrpG* | This study |
| Δ*tolB*/P*hrpX*-GUS | Sp^r^, Δ*tolB* carrying pRG960-P*hrpX* | This study |
| Δ*tolB*/P*gumB*-GUS | Sp^r^, Δ*tolB* carrying pRG960-P*gumB* | This study |
| CΔ*tolB*/P*hrpG*-GUS | Gm^r^Sp^r^, CΔ*tolB* carrying pRG960-P*hrpG* | This study |
| CΔ*tolB*/P*hrpX*-GUS | Gm^r^Sp^r^, CΔ*tolB* carrying pRG960-P*hrpX* | This study |
| CΔ*tolB*/P*gumB*-GUS | Gm^r^Sp^r^, CΔ*tolB* carrying pRG960-P*gumB* | This study |
| *Escherichia coli* | | |
| DH5α | *F^-^ recA hsdR17 (rk^−^, mk^+^) ϕ80lacZ∆M15* | Clontech |
| BL21(DE3) | *F^-^, ompT, hsdSB (rB^-^mB^-^), gal, dcm* | Novagen |
| Yeast | | |
| NYM51 | MATa,*his3-200*,*trp1-901,leu2-3*,*112*,*ade2LYS2::(lexAop)4-HIS3ura3::(lexAop)8-lacZade2::(lexAop)8-ADE2 GAL4* | Clontech |
| **Plasmids** | | |
| pKMS1 | Km^r^, suicide vector derived from pK18mobGII, *sacB*^+^ | (2) |
| pKMS-tolB | Km^r^, a 756-bp fusion cloned in pKMS1 with a 1143-bp deletion comprising 1103-bp of *tolB* coding sequence and 40-bp sequence downstream of translation stop codon | This study |
| pBBR1MCS-5 | Gm^r^, 4.7-kb broad-host range plasmid, *lacZ* | (3) |
| pBB-tolB | Gm^r^, pBBR1MCS-5 harboring a 1500-bp DNA fragment containing *tolB* and promoter region | This study |
| pPR3-N | Amp^r^, DUAL membrane bait vector | Dualsystems Biotech |
| pPR-tolB | Amp^r^, the *tolB* gene with deletion of translation stop codon cloned in pPR3-N at *Sfi*I site | This study |
| pBT3-SET | Km^r^, DUAL membrane prey vector | Dualsystems Biotech |
| pBT-OMP_Xan_ | Km^r^, the *OMP_Xan_* with deletion of translation stop codon cloned in pBT3-SET at *Sfi*I site | This study |
| pET41a (+) | Km^r^, IPTG-inducible expression vector | Novagen |
| pET41-OMP_Xan_ | Km^r^, *OMP_Xan_* gene cloned in pET41a(+) for expressing GST-*OMP_Xan_* fusion | This study |
| pMAL-4X-1 | Amp^r^, an expressing vector with a maltose binding protein (MBP) tag | New England Biolabs |
| pMAL-4X-1-tolB | Amp^r^, *tolB* gene in pMAL-4X-1 for expressing MBP-TolB | This study |
| pRG960 | Sp^r^, broad-host-range vector carrying a promoterless *gusA* gene with start codon | (4) |
| pRG960-P*hrpG* | Sp^r^, a 830-bp *hrpG* promoter region cloned in pRG960 at *Pst*I and *Xma*I sites | This study |
| pRG960-P*hrpX* | Sp^r^, a 873-bp *hrpX* promoter region cloned in pRG960 at *Pst*I and *Xma*I sites | This study |
| pRG960-P*gumB* | Sp^r^, a 94-bp *gumB* promote region cloned in pRG960 at *Bam*HI and *Xma*I sites | This study |

**REFERENCES**

1. Fan X, Guo J, Zhou Y, Zhuo T, Hu X, Zou H. (2018) The *ColRS*-regulated membrane protein gene *XAC1347* is involved in copper homeostasis and *hrp* gene expression in *Xanthomonas citri* subsp. *citri*. *Frontiers in Microbiology*, 9, 1171.
2. Zou L, Li Y, Chen G. (2011) A non-marker mutagenesis strategy to generate poly-hrp gene mutants in the rice pathogen Xanthomonas oryzaepv. oryzicola. Agricultural Science in China 10, 1139*–*1150.
3. Kovach ME, Phillips RW, Elzer PH, Roop R, Peterson KM. (1994) pBBR1MCS: a broad-host-range cloning vector. *Gene,* 166, 175–176.
4. Van den Eede G, Deblaere R, Goethals K, Van Montagu M, Holsters M. (1992) Broad host range and promoter selection vectors for bacteria that interact with plants. *Molecular Plant-Microbe Interactions.* 5, 228–234.
