# Supplementary material for: Xanthomonas citri subsp. citri requires a genus-specific outer membrane protein and TolB to coordinate cell membrane integrity and virulence
Source: Microbiol Spectr. 2025 Jan 16;13(2):e02521-24. doi: 10.1128/spectrum.02521-24 (PMC11792487; doi:10.1128/spectrum.02521-24)
Supplement: Table S3 — Primers used in this study. [file spectrum.02521-24-s0009.docx]

Table S3. Primers used in this study

| **Primer pair** | **Sequence(5’-3’)** | **Cutting sites** | **Description or purpose** | |
| --- | --- | --- | --- | --- |
| **Primers used for molecular cloning**(5' 3') | | | | |
| tolB1.F/tolB1.R | TCGGATCCATGAAAAAACCGCTGC TCTCTAGAACTGGCCGGAACGATCCAGGTC | *Bam*HI-*Xba*I | A 217-bp DNA fragment upstream of *tolB* | |
| tolB2.F/tolB2.R | TCTCTAGAGCCTCCATAGGTACCCCA TCGTCGACTTCTTACTGAGCCGTGTA | *Xba*I-*Sal*I | A 539-bp DNA fragment downstream of *tolB* | |
| CtolB.F/CtolB.R | TCGAATTCGTCGGGCTGAGGCTGCCACT  TGCTCGAGTTAATGCGCGGTTCGGTAAGG | *Eco*RI-*Xho*I | A 1500-bp DNA fragment comprising *tolB* and promoter region | |
| PtolB.F/PtolB.R | TCGGATCCGTCGTCGGGCTGAGGCT  TTCCCGGGACGGCTCGGATCGGGCAGGAA | *Bam*HI-*Xma*I | A 180-bp DNA fragment of *tolB* promoter sequence | |
| Y2HtolB.F/Y2HtolB.R | gtggccattacggccATGAAAAAACCGCTGCGT  TCGGCCGAGGCGGCCATGCGCGGTTCGGTAAGGC | *Sfi*I | *tolB* cloned in pPR3-N at *Sfi*I site | |
| Y2HOMP_Xan_.F/Y2HOMP_Xan_.R | gtggccattacggccACCATTAACAAGCTGCTGATCG  TCGGCCGAGGCGGCCCTTCTTGGCTTCTTCTGCG | *Sfi*I | *OMP_Xan_* cloned in pBT3-SET at *Sfi*I site | |
| GSTOMP_Xan_.F/GSTOMP_Xan_.R | TCGAATTCATGACCATTAACAAGCTGCT  AGGTCGACCTTCTTGGCTTCTTCTGCG | *Eco*RI-*Sal*I | *OMP_Xan_* cloned in pET41a(+) at *Eco*RI-*Sal*I sites | |
| MBPTolB.F/MBPTolB.R | TCGAATTCATGAAAAAACCGCTGCGTTG  AGGTCGACATGCGCGGTTCGGTAAGGC | *Eco*RI-*Sal*I | *tolB* cloned in pET41a(+) at *Eco*RI-*Sal*I sites | |
| PhrpG.F/PhrpG.R | TCCTGCAGGATCATGCCGGTCTCTCT  TTCCCGGGTTAGGCGGCCTTCGCGTGT | *Pst*I-*Xma*I | 830-bp *hrpG* promoter region cloned in pRG960 | |
| PhrpX.F/PhrpX.R | TTCTGCAGCGTCAACAGGAACACCGATC ' TTCCCGGGGCCGGTCTCTCTCTTTGG | *Pst*I-*Xma*I | 873-bp *hrpX* promoter region cloned in pRG960 | |
| PgumB.F/PgumB.R | TTGGATCCACTTTCTGCAGTCCGGTT  TTCTCGAGCCCGGGCGAGCAGATCAACGCCAGGC | *Bam*HI-*Xma*I | 394-bp *gumB* promoter region cloned in pRG960 | |
| **Primers used for qRT-PCR analysis** | | | | |
| *gumB* | ATCGGCGCCAATCTGACCTT; TTGACCATGCGAAACACGAT | | | 97 bp |
| *gumD* | TAACTCGTGGGTCGGCATGAA; GCTGTTCGGTCACGGCAAT | | | 71 bp |
| *hrpG* | GACAGTTCACTGCTGGCATGG; GCGATACCAGGCCAGAATGTT | | | 105 bp |
| *hrpX* | CGACCATGTGTTCTGGCAAG; AATACGCAGCAGGCTACGCA | | | 150 bp |
| *hrpD6* | GGATCCTTCTGGCGAGCGGCTGC; ACGGCATTGAAGTCGTTGCGTGAGG | | | 120 bp |
| *hrcC* | ACCGAGCAACGGAATCTTCGACAGG; TGCAGGATACGCCTACGATGCCGAC | | | 76 bp |
| *gyrA* | TGGCCTCAAGCCTGTGCACCGG; GACGATACGCGCCGACTTGAAG | | | 100 bp |

The 5′ end of each primer contains a restriction enzyme site for cloning into the expression plasmids
